# Interrupted time series study found mixed effects of the impact of the Bavarian smoke-free legislation on pregnancy outcomes

**DOI:** 10.1038/s41598-021-83774-0

**Published:** 2021-02-18

**Authors:** Stephanie Polus, Jacob Burns, Sabine Hoffmann, Tim Mathes, Ulrich Mansmann, Jasper V. Been, Nicholas Lack, Daniela Koller, Werner Maier, Eva A. Rehfuess

**Affiliations:** 1 Institute for Medical Information Processing, Biometry, and Epidemiology – IBE, LMU Munich, Munich, Germany; 2 Pettenkofer School of Public Health, Munich, Germany; 3 Institute for Research in Operative Medicine, Faculty of Health, School of Medicine, Witten/Herdecke University, Cologne, Germany; 4 Division of Neonatology, Department of Paediatrics, Department of Obstetrics and Gynaecology, Department of Public Health, Erasmus MC – Sophia Children’s Hospital, Rotterdam, The Netherlands; 5 German Bavarian Quality Assurance Institute for Medical Care, Munich, Germany; 6 Institute of Health Economics and Health Care Management, Helmholtz Zentrum München – German Research Center for Environmental Health (GmbH), Neuherberg, Germany

**Keywords:** Medical research, Epidemiology, Paediatric research, Health policy, Public health

## Abstract

In 2007 the German government passed smoke-free legislation, leaving the details of implementation to the individual federal states. In January 2008 Bavaria implemented one of the strictest laws in Germany. We investigated its impact on pregnancy outcomes and applied an interrupted time series (ITS) study design to assess any changes in preterm birth, small for gestational age (primary outcomes), and low birth weight, stillbirth and very preterm birth. We included 1,236,992 singleton births, comprising 83,691 preterm births and 112,143 small for gestational age newborns. For most outcomes we observed unclear effects. For very preterm births, we found an immediate drop of 10.4% (95%CI − 15.8, − 4.6%; p = 0.0006) and a gradual decrease of 0.5% (95%CI − 0.7, − 0.2%, p = 0.0010) after implementation of the legislation. The majority of subgroup and sensitivity analyses confirm these results. Although we found no statistically significant effect of the Bavarian smoke-free legislation on most pregnancy outcomes, a substantial decrease in very preterm births was observed. We cannot rule out that despite our rigorous methods and robustness checks, design-inherent limitations of the ITS study as well as country-specific factors, such as the ambivalent German policy context have influenced our estimation of the effects of the legislation.

## Introduction

Over the past two decades, a range of policies and programmes at global, national and regional levels have been designed to reduce the detrimental harms associated with tobacco use^1,2^. There is strong evidence that smoke-free legislation improves adult health outcomes, such as cardiovascular health and mortality from smoking-related illnesses^3^. There is also evidence that smoke-free legislation improves pregnancy outcomes and child health^3,4^. For example, a recent systematic review found reductions in preterm birth rates, perinatal mortality and hospital attendance rates for asthma following smoke-free legislation^4^. However, findings are not fully consistent and based on less rigorous study methods^3,4^, and more rigorous studies are needed to strengthen the evidence base^5^.

Since ratification of the World Health Organization (WHO) Framework Convention on Tobacco Control (FCTC) in 2004, Germany is obliged by international law to implement appropriate measures to reduce and prevent tobacco consumption and second-hand smoke (SHS) exposure^6^. Germany prohibited smoking in the workplace in 2004^7^. In 2007 a national law was passed to protect non-smokers from the harmful consequences of SHS and required the implementation of federal state level legislation to prohibit smoking in public places^8^. Sargent et al.^9^ investigated the short-term effects of the smoke-free legislation on the national level and found a significant decrease in hospital admissions due to acute coronary events after implementation of the smoke-free legislation. No study, however, has assessed the effects of the smoke-free legislation on pregnancy outcomes in the German context.

In this study, we assess the impact of the smoke-free legislation on pregnancy outcomes implemented on 1 January 2008 in Bavaria, Germany’s largest and second most populous state with more than 12.5 million inhabitants and approximately one-sixth of all births in Germany^10^.

## Results

**Figure 1:**
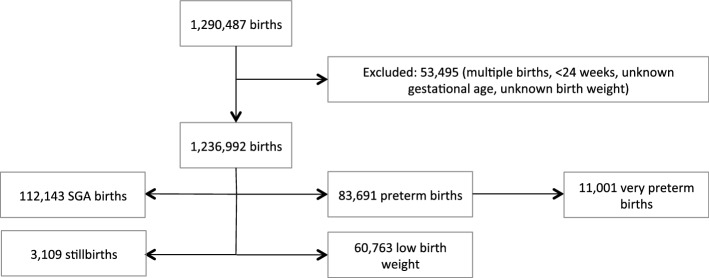
Flow chart of study population and primary and secondary outcomes.

There were 1,290,487 deliveries between 1 January 2005 and 31 December 2016. Due to the standardized data collection process related to Bavarian hospital births, there are no missing data of hospital births. We excluded 53,495 births (4.15%) because inclusion criteria were not met (see Fig. 1). The analysed time series thus included 1,236,992 singleton deliveries, which represents a monthly mean of 8,950 births (range 7,266–10,825). The number of births per month increased steadily over the last 5 years of the study period (see Supplementary Fig. S1 online).

**Figure 2:**
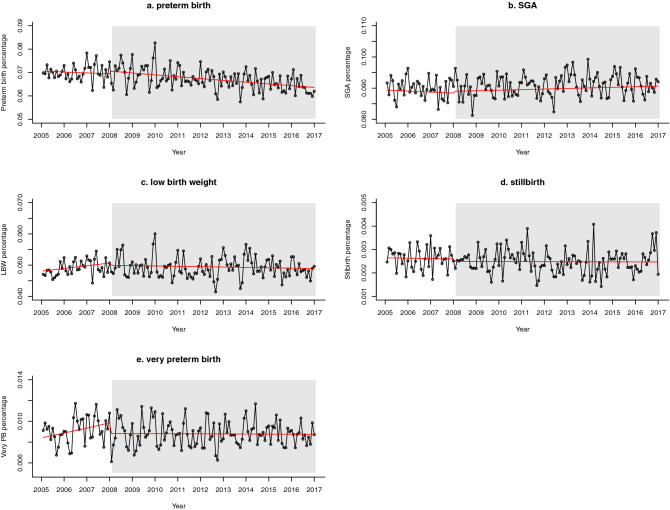
Panel figure presenting time series of pregnancy outcomes in panels (**a**–**e**); dots represent the monthly percentages; white = pre-intervention period, grey = post-intervention period, red line = regression line; SGA = small for gestational age (R version 3.5.1, https://www.r-project.org/).

Overall, there were 83,691 preterm births (< 37 gestational weeks) during the study period. During the same time span there were 112,143 babies born small for gestational age (SGA) (< 10th percentile) and 60,763 born with low birth weight (< 2500 g); 11,001 were very preterm births (< 32 gestational weeks) and 3,109 deliveries were stillbirths (intrauterine death > 500 g). Maternal, newborn and subgroup characteristics are specified in Table 1. Throughout the study period, the outcome rates of all primary and secondary outcomes stayed relatively constant (see Fig. 2). We did not observe a significant underlying trend in primary or secondary outcomes over the study period in the regression models of our main analyses.

**Table 1:** Maternal, newborn and subgroup characteristics by outcome; the numbers represent the number of newborns/mothers with the respective characteristic and the percentage of the births within the outcome group; SES = socio-economic status, BIMD = Bavarian Index of Multiple Deprivation, ranging from least deprived quintile (BIMD1) to most deprived quintile (BIMD5); SGA = small for gestational age, LBW = low birth weight; percent values are rounded to the second decimal place.

Maternal characteristics	Live and non-live births (%) (n = 1,236,992)	Preterm births (%) (n = 83,691)	SGA (%) (n = 112,143)	LBW (%) (n = 60,763)	Stillbirths (%) (n = 3,109)	Very preterm births (%) (n = 11,001)
**Maternal age (years)**						
< 20	19,045 (1.54)	1,594 (1.90)	2,544 (2.27)	1,363 (2.24)	59 (1.90)	249 (2.26)
20–24	129,279 (10.45)	9,311 (11.13)	14,940 (13.32)	7,425 (12.22)	384 (12.35)	1,299 (11.80)
25–29	329,984 (26.67)	21,916 (26.19)	30,763 (27.43)	15,714 (25.86)	745 (23.96)	2,711 (24.64)
30–34	436,317 (35.27)	27,910 (33.35)	36,667 (32.70)	19,499 (32.09)	984 (31.65)	3,486 (31.69)
35–39	258,574 (20.90)	17,695 (21.14)	21,320 (19.01)	12,784 (21.04)	714 (22.97)	2,517 (22.88)
≥ 40	63,793 (5.16)	5,265 (6.29)	5,909 (5.27)	3,978 (6.55)	223 (7.17)	738 (6.71)
Missing	0	0	0	0	0	1 (0.01)
**Parity**						
0	518,743 (41.94)	38,173 (45.61)	60,078 (53.57)	30,119 (45.57)	1,304 (41.94)	4,863 (44.21)
1	408,042 (32.99)	22,734 (27.16)	30,385 (27.09)	15,620 (25.71)	909 (29.24)	2,882 (26.20)
2	185,488 (15.00)	11,873 (14.19)	12,788 (11.40)	7,938 (13.06)	489 (15.73)	1,610 (14.64)
≤ 3	124,691 (10.10)	10,909 (13.03)	8,890 (7.93)	7,083 (11.66)	407 (13.09)	1,645 (14.95)
Missing	28 (0.00)	2 (0.00)	2 (0.00)	3 (0.00)	0 (0.00)	1 (0.01)
**Smoking status**						
Smokers	69,156 (5.59)	6,240 (7.46)	12,974 (11.57)	6,846 (11.27)	248 (7.98)	901 (8.19)
Non-smokers	883,415 (71.42)	57,795 (69.10)	73,264 (65.53)	39,481 (64.98)	2,061 (66.29)	7311 (66.46)
missing	284,421 (22.99)	19,656 (23.49)	25,902 (23.10)	14,434 (18.82)	800 (25.73)	2,789 (25.35)
**SES according to BIMD quintiles**						
BIMD 1 (least deprived)	163,522 (13.22)	10,677 (12.76)	13,569 (12.10)	7,402 (12.18)	404 (12.99)	1,263 (11.48)
BIMD2	147,174 (11.90)	9,823 (11.74)	12,876 (11.48)	6,921 (11.39)	338 (10.87)	1,185 (10.77)
BIMD3	168,880 (13.65)	11,366 (13.58)	15,095 (13.46)	8,222 (13.53)	399 (12.83)	1,413 (12.84)
BIMD4	362,012 (29.27)	23,660 (28.27)	32,558 (29.03)	17,333 (28.53)	895 (28.79)	3,145 (28.59)
BIMD5 (most deprived)	314,219 (25.40)	22,318 (26.67)	30,679 (27.36)	16,647 (27.40)	853 (27.44)	3,098 (28.16)
Missing	81,185 (6.56)	5,847 (6.97)	7,556 (6.74)	4,138 (6.81)	220 (7.08)	897 (8.15)
**Nationality**						
German	999,383 (80.79)	67,699 (80.89)	90,647 (80.83)	49,011 (80.66)	2,367 (76.13)	8,403 (76.39)
Other nationality/missing	237,609 (19.21)	15,992 (19.11)	21,496 (19.17)	11,752 (19.34)	742 (23.87)	2,598 (23.62)
**Infant characteristics**						
*Sex*						
Male	634,111 (51.26)	46,290 (55.31)	57,606 (51.37)	28,863 (47.50)	1,633 (52.52)	6,037 (54.88)
Female	602,881 (48.74)	37,401 (44.69)	54,537 (48.63)	31,900 (52.50)	1,476 (47.48)	4,963 (45.11)
Missing	0	0	0	0	0	1

### Results of main analysis

For the two primary and two out of three secondary outcomes, i.e. preterm birth, SGA, low birth weight and stillbirth, any effect on level changes or slope changes following implementation of the legislation in January 2008 was not statistically significant. We observed small effects with 95% confidence intervals (CIs) that include a decrease as well as an increase of the outcome rates. Results are presented in Table 2 (rate ratios retrieved from exponential beta coefficients) and Fig. 2. These effect estimates can be interpreted as illustrated through the following example: For preterm births, we observed a rate ratio of 1.0163 (95%CI 0.9762, 1.0580) for level change. This represents an immediate relative increase of 1.63% (95%CI − 2.38, 5.80%) in the preterm birth rate (i.e. percentage of total births), which corresponds to a predicted increase in the preterm birth rate from 6.95 to 7.06% from December 2007 to January 2008. The calculated rate ratio of 0.9995 (95%CI 0.9976, 1.0013) for the slope change represents a gradual decrease of 0.05% (95%CI − 0.24, 0.13%) in the preterm birth rate. This corresponds, for example, to a change in the predicted monthly preterm birth rate from 7.031% in May 2008 to a rate of 7.025% in June 2008 and represents an average of two preterm births less every month after implementation of the intervention.

**Table 2:** Estimates of level and slope changes in main analysis of primary and secondary outcomes; SGA = small for gestational age, LBW = low birth weight; All values are rounded to the fourth decimal place.

Outcome	Rate ratio (95%CI)^1^ level change	Rate ratio (95%CI) slope change	Model type
Preterm birth	1.0163 (0.9762, 1.0580)	0.9995 (0.9976, 1.0013)	Negative binomial model with autocorrelation terms
SGA	1.0063 (0.9839, 1.0292)	1.0005 (0.9995, 1.0014)	Poisson model with seasonal dummies and autocorrelation terms
LBW	0.9861 (0.9484, 1.0254)	0.9983 (0.9966, 1.0000)	Negative Binomial with seasonal dummies
Very preterm birth	0.8960 (0.8413, 0.9542), p = 0.0006	0.9954 (0.9928,0.9982), p = 0.0010	Negative binomial model with autocorrelation terms
Stillbirth	0.9583 (0.8165,1.1247)	1.0004 (0.9936, 1.0073)	Poisson model

For the secondary outcome very preterm births we did observe a rate ratio of 0.8960 (95%CI 0.8413, 0.9542) for level change. This represents an immediate relative decrease of very preterm births by 10.40% (95%CI − 15.87, − 4.58%, p = 0.0006), corresponding to a level change from a very preterm birth rate of 0.98% in December 2007 to 0.89% in January 2008. We also observed a rate ratio of 0.9954 (95% 0.9928, 0.9982) for a slope change in very preterm births. This represents a decrease of 0.46% (95%CI − 0.72, − 0.18%, p = 0.0010) in the very preterm birth rate, corresponding to an additional relative decrease from e.g. 0.983% in July 2009 to 0.979% in August 2009, representing four very preterm births less each month.

### Subgroup and sensitivity analyses

**Figure 3:**
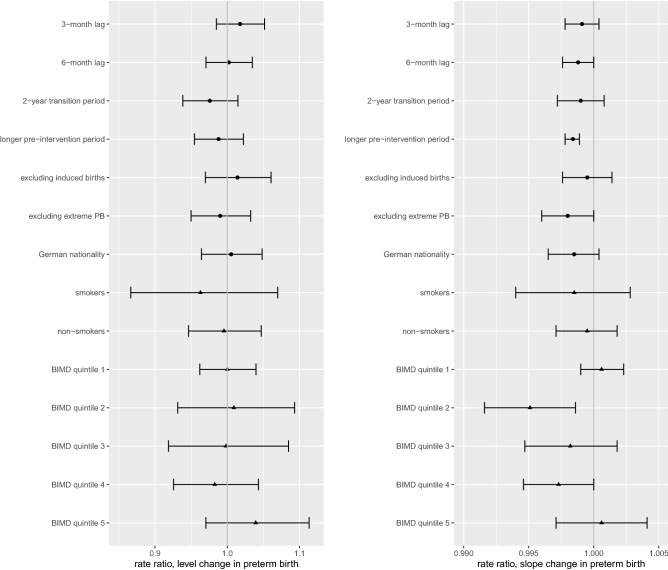
Rate ratios with 95%CIs for level and slope changes of preterm birth, sensitivity (●) and subgroup (▲) analyses (R version 3.5.1, https://www.r-project.org/).

**Figure 4:**
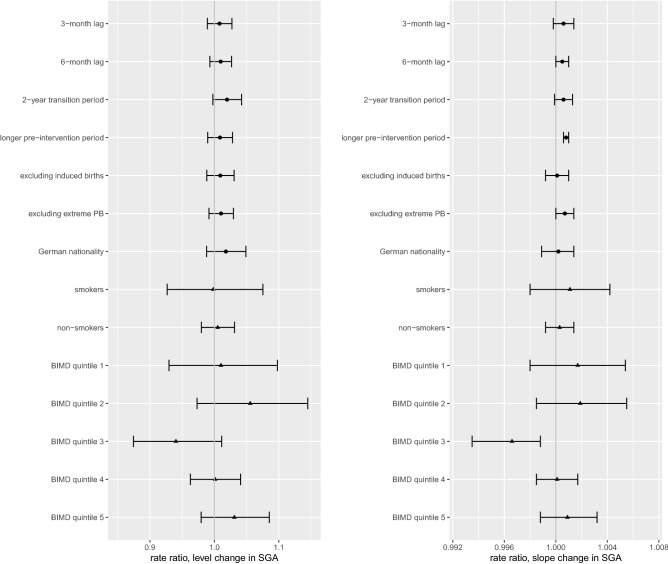
Rate ratios with 95%CIs for level and slope changes of small for gestational age (SGA), sensitivity (●) and subgroup (▲) analyses (R version 3.5.1, https://www.r-project.org/).

Consistent with the main analysis, most of the subgroup and sensitivity analyses showed small effects with confidence intervals suggesting that the effect could be in either direction (see Figs. 3 and 4 and Supplementary Fig. S2-7 online). The detailed results of the sensitivity and subgroup analyses are shown in Supplementary Table S1 online. Active smoking during pregnancy decreased throughout the study period (see Supplementary figure S8 online). For 284,421 deliveries (23%) smoking status information was missing and therefore not included in the subgroup analysis. The mean preterm birth rate was 2.48% (95%CI 2.25–2.70%) and the mean SGA rate 10.52% (95%CI 10.18–10.86%) higher for smokers than for non-smokers (see Supplementary Fig. S9 and S10 online). We did not observe a statistically significant level or slope change in the number of smoking mothers following implementation of the intervention (level = − 1.53, 95%CI − 6.83, 4.06; slope = − 0.18, 95%CI − 0.45, 0.01). We further performed post-hoc sensitivity analyses for our secondary outcome very preterm birth where we could replicate our findings however without reaching statistical significance (see Supplementary Table S2 online).

## Discussion

This study is the first to assess the impact of smoke-free legislation on pregnancy outcomes conducted in Germany. We did not observe clear immediate (level change) or gradual (slope change) effects of the smoke-free legislation on preterm birth, SGA, low birth weight and stillbirth; 95%-confidence intervals surrounding effect estimates suggest that the effect could be in either direction. Our findings were consistent across the majority of sensitivity and subgroup analyses.

We observed statistically significant immediate and gradual reductions for very preterm births. We could replicate these findings in the sensitivity and subgroup analyses especially for smokers; however, here the effects were not statistically significant, probably due to a lack of power considering the much smaller population. Although the preterm birth and SGA rates were higher for the most deprived quintile (BIMD 5) as compared to the least deprived (BIMD 1), consistent with the literature^4,11^, we observed no clear differential effect of the legislation according to SES.

The literature shows mixed effects for the impact of smoke-free legislation on pregnancy outcomes, with a tendency towards a protective effect. Specifically, a recent systematic review and meta-analysis of mostly interrupted time series (ITS) studies performed by Faber et al.^4^ observed an immediate drop (level change) in preterm birth, low birth weight, SGA and very preterm birth, but a gradual decline (slope change) only in SGA and very preterm birth. The effect estimates (risk differences) reported by the included studies were rather small for the outcomes preterm birth, SGA and low birth weight and greater for very preterm birth. In our study we observed, consistent with the meta-analysis in terms of relative effect size, unclear changes in all pregnancy outcomes following the smoke-free legislation except for very preterm birth.

In the absence of studies assessing pregnancy outcomes in Germany, up to now, only one published study employed a time series design to investigate the smoke-free legislation and found a short-term immediate decrease in hospital admissions for acute coronary events^9^. A difference-in-difference study examined short-term effects of the legislation on smoking behaviour using German socio-economic panel (SOEP) data. It found no impact of the legislation in the general population but only for those frequenting bars and restaurants more often^12^.

A previous study conducted in Canada^13^ identified several factors, apart from methodological differences, that may explain the heterogeneous findings across studies assessing the effect of smoke-free legislation on pregnancy outcomes, namely: (i) different policy environments in terms of smoking prevalence and smoking norms, (ii) the presence of existing legislation prior to the smoke-free legislation under investigation, and (iii) differences in policy implementation and enforcement. These factors may contribute to understanding why we did not observe a clearer effect related to the Bavarian smoke-free legislation.

With regards to different policy environments, the smoking prevalence in other countries was similar to the prevalence in Germany, ranging from 18 to 27% among the female population^14–20^. However, Germany, with a prevalence of 27% in 2008–2011 lies at the upper end of this range^21^ and Germany’s efforts in tobacco control have been poor compared to other countries^22–24^. According to the Tobacco Control Ranking Scale 2019^25^, Germany occupies the last rank for successful implementation of tobacco control among 36 mostly European countries. For example, Germany currently is the only EU country, which still allows tobacco advertising on billboards^1^. Tobacco smoking is a well-established risk factor for fetal growth restriction and preterm birth. Indeed, we found large differences between smoking and non-smoking mothers regarding rates of preterm birth and especially SGA. However, we did not observe any drop or gradual decrease of preterm birth or SGA rates in either subgroup following the implementation of the smoke-free legislation. Furthermore, we did not observe changes in smoking rates related to the implementation of the legislation as assumed in our logic model and shown in other studies^14,17,18^. However, caution is merited, as a 5.9% smoking prevalence in our data is very likely an underestimation. On the other hand, the lack of a clear effect could, for example, also be explained by more health-conscious behaviour among pregnant women, who, prior to implementation of the smoke-free legislation, already avoided exposure to second-hand smoke.

Considering pre-existing legislation, as laid out in our logic model, the smoke-free legislation is not the only factor influencing pregnancy outcomes, and we did identify co-interventions and existing legislation prior to implementation of the legislation under investigation that may have subdued a more prevalent effect.

Finally, regarding the role of policy implementation and enforcement, recent studies have shown that the health impact is larger when the smoke-free legislation is more comprehensive^4,26^. While Bavaria has one of the strictest smoke-free legislations within Germany, the legislation lacked supporting interventions, such as an accompanying media campaign or tax increase. Studies in Spain, England and Scotland, where improvements in pregnancy outcomes associated with legislation were observed, also found high compliance with the legislation^17,18,27^. While we do not have data in Bavaria regarding actual enforcement or compliance, it is possible that the unclear effects could be explained by a lack of enforcement or compliance considering Germany’s lacking efforts in tobacco control^12,22,24^.

The ITS study design is prone to certain methodological limitations. The lack of randomization and thus potential confounding make it difficult to definitely attribute causality to the intervention-outcome relationship^5,28^. Beyond this design-inherent limitation, the use of rigorous a priori methods is important.

We used a high quality, large dataset and followed the steps outlined in the tutorial developed by Lopez Bernal et al.^29^ to account for common methodological and conceptual flaws in assessing population level interventions with time series designs^30^. In particular, we took a complex systems approach and used a logic model to conceptualize our study and identified co-interventions and other risk factors prior to conducting our study^31^. We registered a detailed study protocol, in which we defined an impact model, the main statistical analysis, as well as the sensitivity and subgroup analyses. Had we used, for example, our originally planned impact model including the full range of data from 2000 to 2016, we would have come to different conclusions. However, in such a model the pre-intervention slope (and therefore level and slope change) was defined primarily by a major breakpoint in 2004 caused by several co-interventions in this year. This emphasizes the importance of choosing an appropriate impact model, as described by Lopez Bernal et al.^32^.

Our choice of the correct impact model, however, was also associated with several uncertainties. Many aspects of biological processes of pregnancy and especially the exact window of susceptibility of pregnancy to smoking are insufficiently understood^33,34^. Also, the interrupted implementation of the legislation in Bavaria from 2008 to 2010 makes it difficult to identify the exact time at which we can expect to see an effect. Some studies have investigated an immediate onset^18,19^, or even an anticipatory intervention time point^17^ whereas others have used an intervention time point nine months after the actual implementation of the intervention^13^. A study which investigated the smoke-free legislation in the different cantons of Switzerland, found that the more time a mother spent under the smoke-free legislation the fewer were the risks for preterm birth and early-term births^20^.

A further design-inherent weakness of the single-arm ITS study design is the lack of a concomitant, geographical control group^35^. Indeed, a recent study has shown the limitations of single group ITS studies assessing the impact of smoke-free legislation on mortality in Spain where initial protective intervention effects from a single group ITS study were not confirmed after the addition of a comparable geographical control site^36^. Despite careful preparation and consideration, we may have failed to identify important confounders or co-interventions, considering the complexity of the intervention as well as of the system in which it was implemented. Additionally, little concrete guidance on choice of statistical model exists, and determining the ‘best-fit’ model among a range of alternatives remains at least partially arbitrary. Gasparrini et al.^37^ already concluded that the model specifications, among other factors, have a strong impact on the effect estimate when assessing smoke-free legislation on acute myocardial infarction. We aimed, however, to define statistical parameters a priori, where possible, and to comprehensively report modelling choices by publishing our code alongside the manuscript.

## Methods

We applied an ITS study design to assess the association between implementation of the smoke-free legislation and pregnancy outcomes using monthly data from all births in Bavaria between 2005 and 2016. The ITS study design is considered to be one of the best alternatives to assess intervention effectiveness of population-level interventions where randomization is considered infeasible^29,38^. It is increasingly used in the field of healthcare and public health^39,40^. This study design usually draws on routine data collected over time to identify any underlying time trends, and can thereby observe changes after the implementation of an intervention compared to a counterfactual scenario (i.e. a hypothetical scenario in which the intervention was not implemented^41^). We pre-specified the study methods including the main impact model, main analysis, and subgroup and sensitivity analyses in a study protocol (available at www.drks.de, study ID: DRKS00014805).

### Data source

We included aggregated data from a high-quality routine dataset of maternal and neonatal health indicators, collected and managed by the Bavarian Institute for Quality Assurance in hospital care (BAQ). This dataset contains all Bavarian in-hospital births, which constitute about 99% of all births in Bavaria^42^. It provides extensive information retrieved from all hospitals in Bavaria regarding maternal and neonatal demographic and health-related characteristics, clinical management, and pregnancy complications. The data are subjected to a series of formal and contextual plausibility checks^43^.

### Outcomes

Our primary outcomes included preterm birth (< 37 gestational weeks), and SGA (< the 10th percentile, adjusted for gestational age and sex based on Voigt et al.^44^), both measured as the percentage of these outcomes among all births that occurred during a given month. Secondary outcomes included monthly percentages of low birth weight (< 2500 g), very preterm birth (< 32 gestational weeks), and stillbirth (intrauterine death > 500 g).

### Inclusion and exclusion criteria

We included all live and non-live singleton births from 24 until 42 completed weeks of gestation that occurred between January 2005 and December 2016. We excluded pregnancies with multiple births due to their increased risk of preterm birth, low birth weight and other pregnancy complications. We also excluded pregnancies with unknown gestational length and children with unknown birth weight.

### Logic model

**Figure 5:**
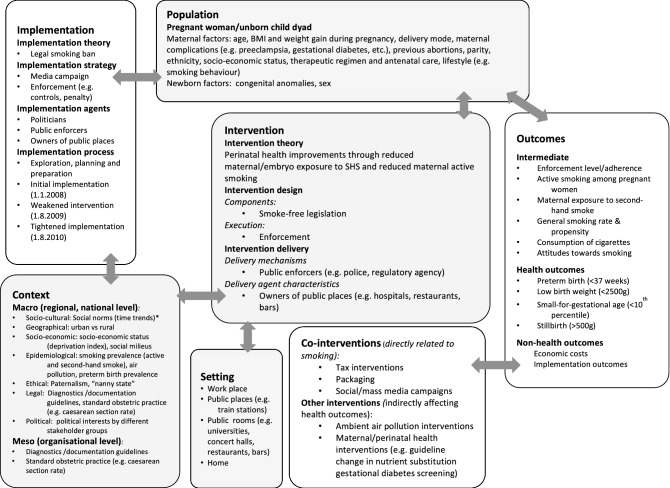
Logic model of the Bavarian smoke-free legislation.

We developed a logic model that describes how the intervention and other factors influence pregnancy outcomes (see Fig. 5) to conceptualise the study and decide on the impact model and statistical analysis. The logic model, derived from literature searches, within-team discussions and expert consultations, provides a structure to help authors address complexity and thus better understand the interactions between the intervention, its implementation and multiple outcomes among a population and context^45,46^.

We identified several co-interventions (i.e. other interventions, measures or policies that occur during the same time period) and other risk factors influencing pregnancy outcomes over the study period, which we describe in detail in our study protocol.

### Impact model

The 16 German federal states are responsible for the implementation of the national law for the protection of non-smokers, and individual state legislation varies in strength (e.g. partial smoke-free laws), as well as in timing of implementation (ranging from August 2007 to July 2008). Bavaria implemented the smoke-free legislation on 1 January 2008. Thereafter, smoking was prohibited in all public buildings and institutions, such as universities, hospitals, retirement and nursing homes, and restaurants and bars^47^. Due to political arguments, however, the Bavarian legislation was loosened on 1 August 2009, and smoking was permitted again in a subset of restaurants, e.g. in restaurants larger than 75 m^2^ mainly serving drinks. Following a referendum, which allowed Bavarian citizens to vote directly for or against more restrictive smoke-free legislation, the legislation was tightened again on 1 August 2010. This iteration additionally banned smoking in beer and event tents^48,49^, making the Bavarian smoke-free legislation one of the strictest in Germany^48^. Violations of smoke-free legislation for smokers as well as event organisers include fines between 5 and 1,000 Euros, but information on enforcement and compliance is lacking.

We hypothesized that the effects of the smoke-free legislation may be detected as an immediate drop (level change) and gradual decline (slope change) in pregnancy outcomes at the first introduction of the smoke-free legislation on 1 January 2008. The changes could be the result of an immediate reduction in maternal exposure to SHS, and/or an immediate reduction in active maternal smoking (in public places and potentially elsewhere). They could also be impacted by longer-term influences on sociocultural norms, affecting smoking behaviours in different settings^50–52^.

Originally, we planned to use data from 2000 to 2016 with equal time periods pre- and post-intervention. Upon visual inspection of the data we identified, however, a series of pronounced changes in outcome rates in the year 2004, during the pre-intervention period, that we were not able to sufficiently account for through adjustments in the analysis. These changes were potentially triggered by the smoking ban at work in August 2004^7^, a major cigarette price increase in September 2004^53^, as well as a documentation change initiated in January 2004. Therefore, we shortened the pre-intervention time period, using data from 2005 to 2016. The main impact model is therefore based on a pre-intervention period from January 2005 to 31 December 2007 and a post-intervention period from 1 January 2008 until 31 December 2016. We report, however, additional sensitivity analyses using data from 2000 to 2016.

### Statistical analyses

We performed a segmented regression analysis using a generalized linear model with log-link for all analyses^29,36^. As the analysed monthly data were not independent from one another, we adjusted for seasonality through the inclusion of monthly dummy variables and/or for autocorrelation through the inclusion of auto-regressive structures in the model. We performed goodness-of-fit tests to decide whether to use a Poisson or a more flexible Negative Binomial model. We scrutinized auto-correlation function (ACF) and partial auto-correlation function (PACF) plots visually and compared Akaike’s information criteria (AIC) to see whether the model performed better after adjustment for seasonality and/or remaining autocorrelation.

The final models were generalized linear models (Poisson or Negative Binomial depending on the outcome) including seasonal dummy variables and/or a random effect term comprising the appropriate autoregressive terms. As we were dealing with count data, we were using the population as an offset variable in order to transform back to rates. The main statistical formula is depicted below:

$$Number\,of\,PB_{t} \sim Poisson\left( {{{\varvec{\uplambda}}}_{t} } \right)\,{\text{or}}\,Number\, of\, PB_{t} \sim\, Negative\, Binomial\left( {{{\varvec{\uplambda}}}_{t} } \right)$$

$${{\varvec{\uplambda}}}_{t} = \left( {\log \left( {Total\, Number \,of\, Birth_{t} } \right)} \right) + \beta_{0} + \beta_{1}\, time_{t} + \beta_{2}\, level_{j} + \beta_{3} \,slope_{jt} + \mathop \sum \limits_{k = 1}^{12} I_{{\left\{ {month\left( t \right) = k} \right\}}}$$

where ${{\varvec{\uplambda}}}_{t}$ is the log of monthly outcome rates measured at each month of observation t, and $time_{t}$ is a continuous variable modelling each month since January 2005 (1,2,3…–145), $level_{j}$ a binary predictor for the legislation, which is modelled as 0 in the pre-legislation time period (January 2005–December 2007) and 1 in the post-legislation period (January 2008–December 2016). $slope_{jt}$ is an interaction term of the legislation with time. In this model $\beta_{0}$ represents the baseline outcome rate, $\beta_{1}$ the change in outcome rate per one unit increase in time (month) (i.e. the underlying pre-legislation trend), $\beta_{2}$ the level change in outcome following the legislation and $\beta_{3}$ the slope change in outcome following the legislation.

We performed data management with SAS software version 9.4^54^, and the analyses using R^55^. The complete R code for the main statistical analysis is available in the Supplementary Information online.

### Subgroup and sensitivity analyses

We specified a series of sensitivity and subgroup analyses a priori for the primary outcomes preterm birth and SGA (see Table 3). We further performed post-hoc sensitivity analyses for the secondary outcome very preterm birth to verify the findings in the main analysis.

**Table 3:** Sensitivity and subgroup analysis descriptions.

Sensitivity analyses
**Varying time lags** We tested different intervention time points with three and six month time lags to assess if our assumption that smoking can affect pregnancy outcomes at any stage during pregnancy was correct
**Excluding transition period** We excluded the data from 1 January 2008 (when the first smoking ban was implemented) until 1 August 2010 (when the smoking ban was reinstated) to compare the time period prior to the first ban to the period after implementation of the tightened ban
**Including a longer pre-intervention period (2000–2016) (post-hoc)** We analysed the originally planned impact model with a study period from 2000 to 2016
**Excluding induced births** We analysed spontaneous preterm births only, as smoking is associated with the spontaneous preterm onset of labour due to the inflammatory responses it triggers and higher risk of intrauterine uterine infections (Goldenberg, Culhane, Iams, & Romero, 2008). We could thereby also account for the potential effects of the introduction of gestational diabetes screening in 2011^56^, which is associated with induced preterm births
**Excluding preterm infants at the border of viability** We excluded infants born between 24 and 27 completed gestational weeks and only assessed infants born between 28 and 36 gestational weeks to rule out any effect of changes in data documentation practices after implementation of the guidelines on premature infants on the border of viability^57^
**Including only mothers of German nationality** We excluded all mothers born outside of Germany to rule out any effect of the recent increase in refugees starting in 2014 in Germany

Subgroup analyses
**Smoking status** We tested if outcomes rates differed between actively smoking and non-smoking mothers (see Mackay et al.^17^). Smoking, as reported by the mother, is recorded when registering in hospital. We further assessed post-hoc whether the smoking rates differed after implementation of the smoke-free legislation
**Socio-economic status (SES)** We wanted to assess the impact of tobacco control policies on marginalised populations and assessed whether the legislation had a different effect on different socio-economic groups. Individual-level SES data were not available, and we thus used the area-level Bavarian Index of Multiple Deprivation (BIMD) as a proxy for individual SES^58,59^. We therefore assigned each mother a BIMD quintile based on the postal code of her residential address. We then performed subgroup analyses according to each BIMD quintile

### Ethics statement

As we use anonymous, routinely collected data, separate ethics approval was not required for this study, as confirmed by a waiver obtained from the ethics commission of the LMU Munich. The Bavarian Institute for Quality Assurance in hospital care (BAQ) approved the use of the data.

## Supplementary information

Supplementary Information.

## Data Availability

The data that support the findings of this study are available from the Bavarian Institute for Quality Assurance in hospital care (BAQ) but restrictions apply to the availability of these data, which were used under license for the current study, and so are not publicly available. Data are however available from the authors upon reasonable request and with permission of BAQ.
