# Supplementary material for: Interrupted time series study found mixed effects of the impact of the Bavarian smoke-free legislation on pregnancy outcomes
Source: Sci Rep. 2021 Feb 18;11:4209. doi: 10.1038/s41598-021-83774-0 (PMC7892567; doi:10.1038/s41598-021-83774-0)
Supplement: Supplementary file 1 — Supplementary Information. [file 41598_2021_83774_MOESM1_ESM.docx]

**Supplementary Information**

**Interrupted time series study found mixed effects of the impact of the Bavarian smoke-free legislation on pregnancy outcomes**

Stephanie Polus ^*1,2^, Jacob Burns^1,2^, Sabine Hoffmann ^1,2^, Tim Mathes^3^, Ulrich Mansmann^1,2^, Jasper V Been^4^, Nicholas Lack^5^, Daniela Koller^1,2^, Werner Maier^6^, Eva A Rehfuess^1,2^

*corresponding author

^1^ Institute for Medical Information Processing, Biometry, and Epidemiology – IBE, LMU Munich, Marchioninistr. 17, 81377 Munich, Germany

^2^ Pettenkofer School of Public Health, Munich, Germany

^3^ Institute for Research in Operative Medicine, Faculty of Health, School of Medicine, Witten/Herdecke University, Cologne, Germany

^4^ Division of Neonatology, Department of Paediatrics, Department of Obstetrics and Gynaecology, Department of Public Health, Erasmus MC – Sophia Children’s Hospital, Rotterdam, Netherlands

^5^ German Bavarian Quality Assurance Institute for Medical Care, Munich, Germany

^6^ Institute of Health Economics and Health Care Management, Helmholtz Zentrum München – German Research Center for Environmental Health (GmbH), Neuherberg, Germany

Table of Contents

Figure S1 Number of births 2005-2016 2

Figure S2 Preterm birth rate 2000-2016 including regression line 2

Figure S3 SGA rate 2000-2016 including regression line 2

Figure S4 Preterm birth rate by highest (BIMD 1) and lowest (BIMD 5) SES, 2005-2016 3

Figure S5 SGA rate by highest (BIMD 1) and lowest (BIMD 5) SES, 2005-2016 3

Figure S6 Regression lines preterm birth rates according to SES (BIMD quintile) 4

Figure S7 Regression lines SGA rates according to SES (BIMD quintile) 4

Figure S8 Number of smoking mothers 2005-2016 4

Figure S9 Preterm birth rates among smoking and non-smoking mothers, 2005-2016 5

Figure S10 SGA rates smoking and non-smoking mothers, 2005-2016 5

Table S1 Estimates of changes in level and slope in subgroup and sensitivity analyses of primary outcomes. 6

Table S2 Post-hoc sensitivity analyses very preterm birth 7

R Script of main analysis 8

### Figure S1 Number of births 2005-2016


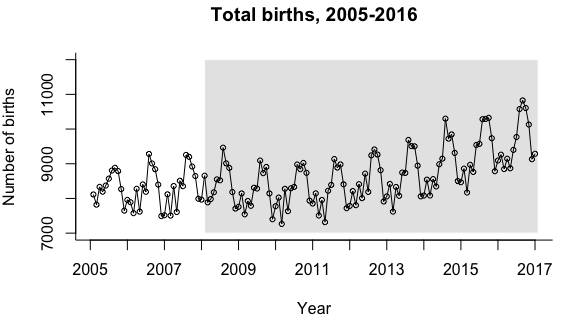


### Figure S2 Preterm birth rate 2000-2016 including regression line


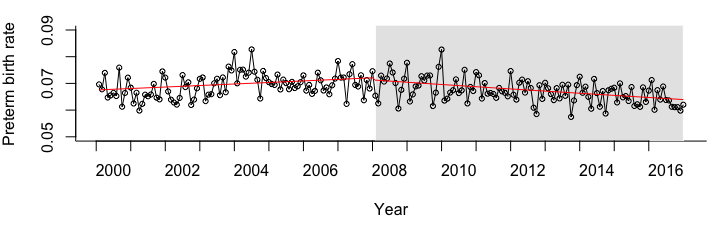


### Figure S3 SGA rate 2000-2016 including regression line


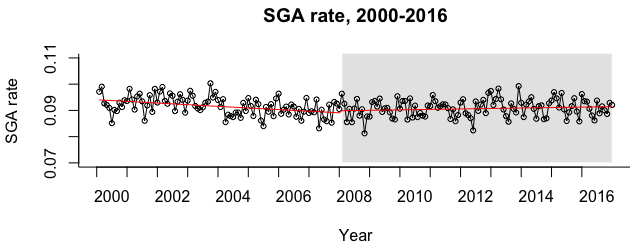


### Figure S4 Preterm birth rate by highest (BIMD 1) and lowest (BIMD 5) SES, 2005-2016

**
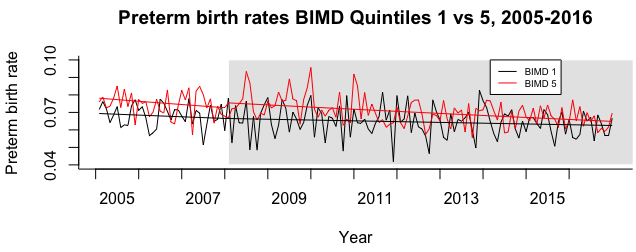
**

### Figure S5 SGA rate by highest (BIMD 1) and lowest (BIMD 5) SES, 2005-2016


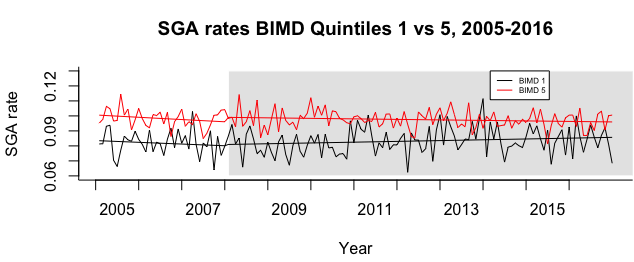


### Figure S6 Regression lines preterm birth rates according to SES (BIMD quintile)


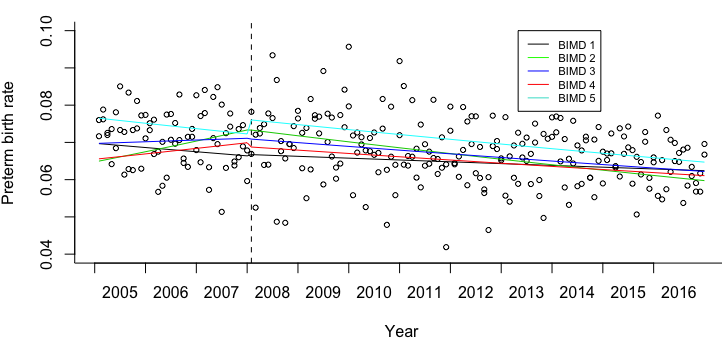


### Figure S7 Regression lines SGA rates according to SES (BIMD quintile)


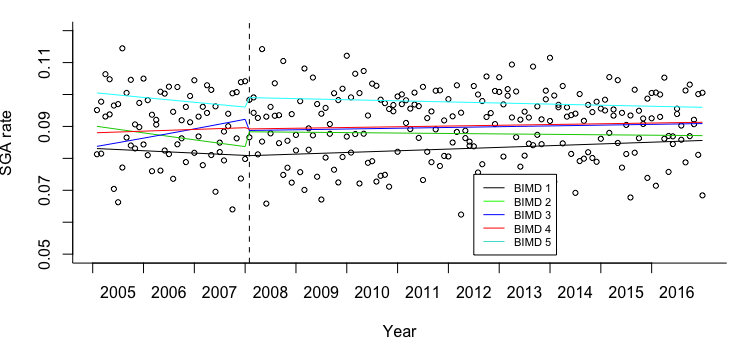


### Figure S8 Number of smoking mothers 2005-2016


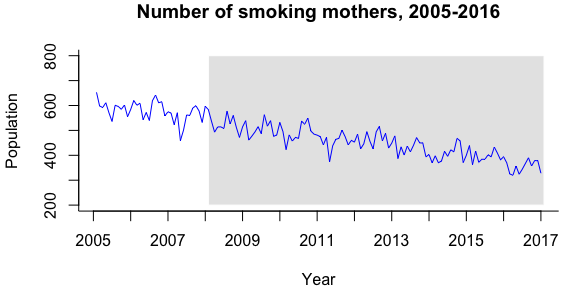


### Figure S9 Preterm birth rates among smoking and non-smoking mothers, 2005-2016

**
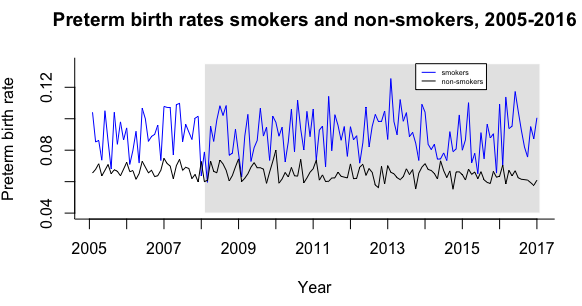
**

### Figure S10 SGA rates smoking and non-smoking mothers, 2005-2016


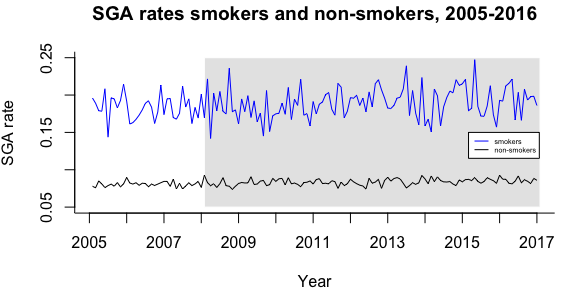


### Table S1 Estimates of changes in level and slope in subgroup and sensitivity analyses of primary outcomes.

| **Outcome** | **Exponential level coefficient (95%CI)** | **Exponential slope coefficient (95%CI)** | **Model type (and R package used)** |
| --- | --- | --- | --- |
| **Sensitivity analyses** | | | |
| **3-month lag after legislation implementation** | | | |
| Preterm birth | 1.0177 (0.9849,1.0516) | 0.9991 (0.9978,1.0004) | Seasonally adjusted Negative Binomial model (glm) |
| SGA | 1.0080 (0.9891,1.0273) | 1.0006 (0.9998, 1.0014) | Poisson model (glarma) with seasonal dummies and autocorrelation terms |
| **6-month lag after legislation implementation** | | | |
| Preterm birth | 1.002 (0.9705, 1.0346) | 0.9988 (0.9976,1.0000), p=0.03947 | Seasonally adjusted Negative Binomial model (glm) |
| SGA | 1.0097 (0.9930, 1.0267) | 1.0005 (1.0000, 1.0010) | Poisson model (glarma) with seasonal dummies and autocorrelation terms |
| **Excluding data of a 2-year transition period after legislation implementation** | | | |
| Preterm birth | 0.9758 (0.9384, 1.0147) | 0.9990 (0.9972,1.0008) | Negative binomial model (glarma) with autocorrelation terms |
| SGA | 1.0197 (0.9977,1.0423) | 1.0006 (0.9999, 1.0013) | Poisson model (glarma) with autocorrelation terms |
| **Including a longer pre-intervention period (2000-2016) (post-hoc)** | | | |
| Preterm birth | 0.9879 (0.9546, 1.0223) | 0.9984 (0.9978, 0.9989), p=0.0000 | Negative Binomial model (glarma) with seasonal dummies and autocorrelation terms |
| SGA | 1.0087 (0.9897,1.0281) | 1.0008 (1.0006, 1.0010), p=0.0000 | Poisson model (glarma) with seasonal dummies & autocorrelation terms |
| **Excluding induced births** | | | |
| Preterm birth | 1.0141 (0.9697, 1.0605) | 0.9995 (0.9976, 1.0014) | Negative binomial model (glm) with seasonal dummies |
| SGA | 1.0092 (0.9882,1.0306) | 1.0001 (0.9992,1.0010) | Poisson model (glarma) with autocorrelation terms |
| **Excluding preterm infants at the border of viability** | | | |
| Moderate preterm birth | 0.9902 (0.9498, 1.0323) | 0.9980 (0.9960,1.0000), p=0.0384 | Negative binomial model (glarma) with autocorrelation terms |
| SGA | 1.0104 (0.9916, 1.0296) | 1.0007 (1.0000,1.0014) | Poisson model (glarma) with autocorrelation terms |
| **Including only mothers of German nationality** | | | |
| Preterm birth | 1.0053 (0.9642, 1.0482) | 0.9985 (0.9965, 1.0004) | Negative binomial model (glarma) with autocorrelation terms |
| SGA | 1.0179 (0.9879, 1.0489) | 1.0002 (0.9989, 1.0014) | Unadjusted Poisson model (glm) |
| **Subgroup analyses** | | | |
| **Smoking status** | | | |
| Preterm birth | Smokers:  0.9627 (0.8664,1.0698) | 0.9985 (0.9940,1.0028) | Unadjusted Poisson model (glm) |
|  | Non-smokers:  0.9954 (0.9464,1.0469) | 0.9995 (0.9971,1.0018) | Negative binomial model (glarma) with autocorrelation terms |
| SGA | Smokers:  0.9983 (0.9267, 1.0753) | 1.0011 (0.9980,1.0042) | Unadjusted Poisson model (glm) |
|  | Non-smokers:  1.0052 (0.9798, 1.0313) | 1.0003 (0.9992, 1.0014) | Poisson model (glarma) with autocorrelation terms |
| **Socio-economic status – Preterm birth** | | | |
| **BIMD quintile 1 (highest SES)** | 1.0001 (0.9619, 1.0397) | 1.0006 (0.9990, 1.0023) | Poisson model (glarma) with autocorrelation terms |
| **BIMD quintile 2** | 1.0090 (0.9314, 1.0932) | 0.9951 (0.9916, 0.9986), p=0.0061 | Poisson model (glarma) with autocorrelation terms |
| **BIMD quintile 3** | 0.9982 (0.9186, 1.0847) | 0.9982 (0.9947, 1.0018) | Unadjusted Poisson model |
| **BIMD quintile 4** | 0.9826 (0.9256, 1.0430) | 0.9973 (0.9946, 1.0000), p=0.04 | Poisson model (glarma) with autocorrelation term |
| **BIMD quintile 5 (lowest SES)** | 1.0393 (0.9703, 1.1132) | 1.0006 (0.9971, 1.0041) | Negative binomial model (glarma) with autocorrelation terms |
| **Socio-economic status – SGA** | | | |
| **BIMD quintile 1 (highest SES)** | 1.0102 (0.9295, 1.0979) | 1.0017 (0.9980, 1.0054) | Poisson model (glarma) with autocorrelation terms |
| **BIMD quintile 2** | 1.0556 (0.9731, 1.1450) | 1.0019 (0.9985, 1.0055) | Unadjusted Poisson model |
| **BIMD quintile 3** | 0.9403 (0.8743, 1.0114) | *0.9966 (0.9935, 0.9988), p=0.03* | Poisson model (glarma) with autocorrelation terms |
| **BIMD quintile 4** | 1.0010 (0.9628, 1.0407) | 1.0001 (0.9985, 1.0017) | Poisson model (glarma) with autocorrelation terms |
| **BIMD quintile 5 (lowest SES)** | 1.0310 (0.9794, 1.0853) | 1.0009 (0.9988, 1.0032) | Unadjusted Poisson model |

### Table S2 Post-hoc sensitivity analyses very preterm birth

| **Sensitivity analyses** | **Exponential level coefficient (95%CI)*** | **Exponential slope coefficient (95%CI)** | **Model type (and R package used)** |
| --- | --- | --- | --- |
| 3 month lag | 0.9077 (0.8291, 0.9938), p= 0.0364 | 0.9962 (0.9922, 1.0002) | Negative binomial model (glarma) with autocorrelation terms |
| 6 month lag | 0.9080 (0.8287,0.9949), p=0.0384 | 0.9962 (0.9922, 1.0002) | Negative binomial model (glarma) with autocorrelation terms |
| 2-year transition period | 0.9745 (0.9357, 1.0149) | 0.9985 (0.9961,1.0001) | Negative binomial model (glarma) with autocorrelation terms |
| Longer time period 2000-2016 | 0.9967 (0.9051, 1.0329) | 0.9993 (0.9982, 1.0004) | Negative binomial model (glarma) with autocorrelation terms |
| Excluding induced births | 0.8667 (0.7297, 1.0308) | 0.9954 (0.9880,1.0028) | Negative binomial model (GLM) with seasonal adjustment |
| Including only mothers of German nationality | 0.9268 (0.8250,1.0412) | 0.9957 (0.9906,1.0007) | Negative binomial model (glarma) with autocorrelation terms |
| Smokers | 0.8161 (0.6318, 1.0540) | 0.9959 (0.9851,1.0007) | Poisson model (glarma) with autocorrelation terms |
| Non-smokers | 0.9093 (0.8134,1.0164) | 0.9956 (0.9908,1.0004) | Unadjusted negative binomial model (glm) |
| BIMD 1 | 0.9365 (0.7316,1.1987) | 0.9963 (0.9856,1.0071) | Unadjusted Poisson model |
| BIMD 2 | 1.0361 (0.7958,1.349) | 0.9938 (0.9822,1.0055) | Unadjusted Poisson model |
| BIMD 3 | 1.0595 (0.8311,1.3508) | 1.0037 (0.9934,1.0141) | Unadjusted Poisson model |
| BIMD 4 | 0.8845 (0.7690,1.0174) | 0.9935 (0.8637,1.1473) | Negative binomial model (glarma) with autocorrelation terms |
| BIMD 5 | 0.8992 (0.7869,1.0275) | 0.9979 (0.9909,1.0049) | Negative binomial model (glarma) with autocorrelation terms |

### R Script of main analysis

#load necessary packages

library(foreign)

library(dplyr)

library(MASS)

library(glarma)

source("/Users/likTestsNEW.R") #in the glarma package this was necessary to be able to use the package properly

source("/Users/summary.glarmaNEW.R") #sources kindly provided by William Duinsmuir

#retrieve data

smoke <- read.xport("/smoke.xpt")

#rename variables

smoke$year <- smoke$YEAR

smoke$YEAR <- NULL

smoke$month <- smoke$MONTH

smoke$MONTH <- NULL

smoke$pb <- smoke$PB

smoke$PB <- NULL

smoke$vpb <- smoke$VPB

smoke$VPB <- NULL

smoke$sga <- smoke$SGA

smoke$SGA <- NULL

smoke$lbw <- smoke$LBW

smoke$LBW <- NULL

smoke$still <- smoke$STILL

smoke$STILL <- NULL

smoke$n <- smoke$N

smoke$N <- NULL

smoke$t <- smoke$T

smoke$T <- NULL

#We delete the data smaller 2005 and bigger than 2016

smoke <- smoke %>% filter(year >=2005 & year != 2017)

#We set time variabe t at new dataset start

smoke$t <- 1:nrow(smoke)

##############

#create time variables

################

cp <- 37 #set intervention time point on 1 January 2008

smoke$level <- ifelse(smoke$t<cp, 0, 1)

smoke$slope <- c(rep(0, cp-1), 1:(145-cp))

#plot whole time series

# start the plot, excluding the points and the x-axis

plot(smoke$pb,type="n",ylim=c(0.05,0.09),xlab="Year", ylab="Preterm birth rate",

bty="l",xaxt="n")

# Add line indicating the policy changes

abline(v=37,lty=2)

# plot the observed rate for intervention period

points(smoke$pb,cex=0.7, col="red") #oder mit points

lines(smoke$pb,cex=0.7, col="red")

#specify the x-axis (i.e. time units)

axis(1,at=0:12*12,labels=F)

axis(1,at=0:12*12,tick=F,labels=2005:2017)

# add a title

title("Preterm rate, 2005-2016")

#plot numbers

plot(smoke$pbn,type="n",ylim=c(450,700),xlab="Year", ylab="Preterm birth rate",

bty="l",xaxt="n")

# Add line indicating the policy changes

abline(v=37,lty=2)

# plot the observed rate for intervention period

points(smoke$pbn,cex=0.7) #oder mit points

lines(smoke$pbn,cex=0.7)

#specify the x-axis (i.e. time units)

axis(1,at=0:12*12,labels=F)

axis(1,at=0:12*12,tick=F,labels=2005:2017)

# add a title

title("Preterm rate, 2005-2016")

#create offset for model

smoke$pbn <- smoke$pb * smoke$n

smoke$logn <- log(smoke$n)

head(smoke)

#first try of glm model

model1 <- glm(pbn ~ offset(logn) + t + level + slope, data=smoke, family = poisson())

summary(model1)

# goodness of fit test: if test is significant, model fit is not good

1 - pchisq(summary(model1)$deviance,

summary(model1)$df.residual)

#negative binomial model

model2 <- glm.nb(pbn ~ offset(logn) + t + level + slope, data=smoke, link=log)

summary(model2)

1 - pchisq(summary(model2)$deviance,

summary(model2)$df.residual) #better fit

par(mfrow=c(1, 2))

acf(residuals(model2))

acf(residuals(model2),type='partial') #looks like months/seasonality play more of a role

#plot residuals

plot(smoke$t, residuals(model2), type='o', pch=16)

#adjust with monthly dummies

smoke$jan <- ifelse(smoke$month==1, 1,0)

smoke$feb <- ifelse(smoke$month==2, 1,0)

smoke$mar <- ifelse(smoke$month==3, 1,0)

smoke$apr <- ifelse(smoke$month==4, 1,0)

smoke$may <- ifelse(smoke$month==5, 1,0)

smoke$jun <- ifelse(smoke$month==6, 1,0)

smoke$jul <- ifelse(smoke$month==7, 1,0)

smoke$aug <- ifelse(smoke$month==8, 1,0)

smoke$sep <- ifelse(smoke$month==9, 1,0)

smoke$oct <- ifelse(smoke$month==10, 1,0)

smoke$nov <- ifelse(smoke$month==11, 1,0)

smoke$dec <- ifelse(smoke$month==12, 1,0)

model3 <- glm.nb(pbn ~ offset(logn) + t + level + slope + jan + feb + mar + apr + may +

jun + jul + aug + oct + nov + dec, data=smoke, link=log, maxit=300)

summary(model3)

#plot residuals

plot(smoke$t, residuals(model3), type='o', pch=16)

#goodness of fit test

1 - pchisq(summary(model3)$deviance,

summary(model3)$df.residual) #looks good

#look for remaining autocorrelation

par(mfrow=c(1, 2))

acf(residuals(model3))

acf(residuals(model3),type='partial') #still autocorrelation present

#glarma model

library(glarma)

source("/Users/stephie/LRZ Sync+Share/ITS PhD/likTestsNEW.R")

source("/Users/stephie/LRZ Sync+Share/ITS PhD/summary.glarmaNEW.R")

predictors <- cbind(intercept <-(rep(1, dim(smoke)[1])),

slope <-smoke$slope, level <- smoke$level, t <-smoke$t)

colnames(predictors) <- c("intercept", "slope", "level", "t")

model5 <- glarma(smoke$pbn, predictors, offset = smoke$logn, type = "NegBin",

phiLags = c(9,12,21)) #final model

par(mfrow=c(1, 2))

acf(residuals(model5))

acf(residuals(model5),type='partial')#looks good

#plot regression line

dev.off()

# start the plot, excluding the points and the x-axis

plot(smoke$pb,type="n",ylim=c(0.05,0.09),xlab="Year", ylab="Preterm birth percentage",

bty="l",xaxt="n")

# Add line indicating the policy changes

rect(37,0.05,145,0.09,col=grey(0.9),border=F)

# plot the observed rate for intervention period

points(smoke$pb,cex=0.7)

lines(smoke$pb,cex=0.7)

#specify the x-axis (i.e. time units)

axis(1,at=0:12*12,labels=F)

axis(1,at=0:12*12,tick=F,labels=2005:2017)

# add a title

title("a. Preterm birth")

#plot regression line with glarma model

coef(model5)

predict <- exp(smoke$logn + coef(model5)$beta[1]

+ smoke$t*coef(model5)$beta[4]

+ smoke$level*coef(model5)$beta[3]

+ smoke$slope*coef(model5)$beta[2])

lines(predict/smoke$n,col=2)

#####################################################

#SGA

#####

# start the plot, excluding the points and the x-axis

plot(smoke$sga,type="n",ylim=c(0.08,0.11),xlab="Year", ylab="SGA rate",

bty="l",xaxt="n")

# Add line indicating the policy changes

abline(v=37,lty=2)

# plot the observed rate for intervention period

points(smoke$sga,cex=0.7, col="red") #oder mit points

lines(smoke$sga,cex=0.7, col="red")

#specify the x-axis (i.e. time units)

axis(1,at=0:12*12,labels=F)

axis(1,at=0:12*12,tick=F,labels=2005:2017)

# add a title

title("SGA rate, 2005-2016")

#create offset for model

smoke$sgan <- smoke$sga * smoke$n

head(smoke)

model1 <- glm(sgan ~ offset(logn) + t + level + slope, data=smoke, family = poisson())

summary(model1)

# If test is significant, model fit is not good

1 - pchisq(summary(model1)$deviance,

summary(model1)$df.residual) #good fit

par(mfrow=c(1, 2))

acf(residuals(model1))

acf(residuals(model1),type='partial') #again seasonality?

#plot residuals

plot(smoke$t, residuals(model1), type='o', pch=16)

#model with monthly dummies

model2 <- glm(sgan ~ offset(logn) + t + level + slope + jan + feb + mar + apr + may

+ jun + jul + aug + oct + nov + dec,

data=smoke, family=poisson)

summary(model2)

par(mfrow=c(1, 2))

acf(residuals(model4))

acf(residuals(model4),type='partial')

#glarma

predictors <- cbind(intercept <-(rep(1, dim(smoke)[1])),

slope <-smoke$slope, level <- smoke$level, t <-smoke$t,

jan <- smoke$jan, feb <- smoke$feb,mar <- smoke$mar,

apr <- smoke$apr, may <- smoke$may, jun <- smoke$jun,

jul <- smoke$jul, aug <- smoke$aug, oct <- smoke$oct,

nov <- smoke$nov, dec <- smoke$dec)

colnames(predictors) <- c("intercept", "slope", "level", "t", "jan", "feb", "mar",

"apr", "may", "jun", "jul", "aug", "oct", "nov", "dec")

model4 <- glarma(smoke$sgan, predictors, offset = smoke$logn, type = "Poi") #seasonal model does not look like a good fit

summary(model4)

par(mfrow=c(1, 2))

acf(residuals(model4))

acf(residuals(model4),type='partial')

#try glarma with autocorrelation

model4_10 <- glarma(smoke$sgan, predictors, offset = smoke$logn, type = "Poi", phiLags = c(4,10)) #final model

summary(model4_10)

acf(residuals(model4_10))

acf(residuals(model4_10),type='partial') #looks ok with monthly dummies and AR structure

#plot regression line

plot(smoke$sga,type="n",ylim=c(0.08,0.11),xlab="Year", ylab="SGA percentage",

bty="l",xaxt="n")

# Add line indicating the policy changes

rect(37,0.08,145,0.11,col=grey(0.9),border=F)

#abline(v=37,lty=2)

# plot the observed rate for intervention period

points(smoke$sga,cex=0.7)

lines(smoke$sga,cex=0.7)

#specify the x-axis (i.e. time units)

axis(1,at=0:12*12,labels=F)

axis(1,at=0:12*12,tick=F,labels=2005:2017)

# add a title

title("b. SGA")

#print regression line

coef(model4_10)

predict <- exp(smoke$logn + coef(model4_10)$beta[1]

+ smoke$t*coef(model4_10)$beta[4]

+ smoke$level*coef(model4_10)$beta[3]

+ smoke$slope*coef(model4_10)$beta[2])

lines(predict/smoke$n,col=2)

#######################################

#lbw

plot(smoke$lbw,type="n",ylim=c(0.04,0.07),xlab="Year", ylab="LBW percentage",

bty="l",xaxt="n")

# shade the post intervention period grey

rect(37,0.04,145,0.07,col=grey(0.9),border=F)

# plot the observed rate for intervention period

points(smoke$lbw,cex=0.7)

lines(smoke$lbw,cex=0.7)

#specify the x-axis (i.e. time units)

axis(1,at=0:12*12,labels=F)

axis(1,at=0:12*12,tick=F,labels=2005:2017)

# add a title

title("c. LBW")

head(smoke)

#create offset for model

smoke$lbwn <- smoke$lbw * smoke$n

head(smoke)

model2 <- glm.nb(lbwn ~ offset(logn) + t + level + slope, data=smoke, link=log)

summary(model2)

# goodness of fit test

1 - pchisq(summary(model2)$deviance,

summary(model2)$df.residual) #neg bin model is the better fit

par(mfrow=c(1, 2))

acf(residuals(model2))

acf(residuals(model2),type='partial')

#try with seasonal adjustment

model3 <- glm.nb(lbwn ~ offset(logn) + t + level + slope + jan + feb + mar + apr + may

+ jun + jul + aug + oct + nov + dec, data=smoke, link=log, maxit = 300)

summary(model3)

acf(residuals(model3))

acf(residuals(model3),type='partial') #looks good with seasonal adjustment

#plot regression line

#pred <- predict(model3,type="response")

plot(smoke$lbw,type="n",ylim=c(0.04,0.07),xlab="Year", ylab="LBW rate",

bty="l",xaxt="n")

# Add line indicating the policy changes

#abline(v=37,lty=2)

# shade the post intervention period grey

rect(37,0.04,145,0.07,col=grey(0.9),border=F)

# plot the observed rate for intervention period

points(smoke$lbw,cex=0.7)

lines(smoke$lbw,cex=0.7)

#specify the x-axis (i.e. time units)

axis(1,at=0:12*12,labels=F)

axis(1,at=0:12*12,tick=F,labels=2005:2017)

# add a title

title("LBW rate")

#without seasonality visible:

newdata <- smoke

head(newdata)

newdata[(14:25)] <- 0

#plot new data without seasonality

pred3 <- predict(model3,newdata=newdata)

coef(model3)

mean_months <- mean(coef(model3)[5:15])

lines(exp(pred3 + mean_months)/smoke$n,col="red")

####################################

#stillbirth

##################

#plot stillbirth rate

plot(smoke$still,type="n",ylim=c(0.001,0.005),xlab="Year", ylab="stilbirth percentage",

bty="l",xaxt="n")

# Add line indicating the policy changes

#abline(v=37,lty=2)

rect(37,0.001,145,0.005,col=grey(0.9),border=F)

# plot the observed rate for intervention period

points(smoke$still,cex=0.7)

lines(smoke$still,cex=0.7)

#specify the x-axis (i.e. time units)

axis(1,at=0:12*12,labels=F)

axis(1,at=0:12*12,tick=F,labels=2005:2017)

# add a title

title("d. stillbirth")

#create offset for model

smoke$stilln <- smoke$still * smoke$n

head(smoke)

model1 <- glm(stilln ~ offset(logn) + t + level + slope, data=smoke, family = poisson())

summary(model1)

# If test is significant, model fit is not good

1 - pchisq(summary(model1)$deviance,

summary(model1)$df.residual) #fits

par(mfrow=c(1, 2))

acf(residuals(model1))

acf(residuals(model1),type='partial') #looks already good

#plot regression line

plot(smoke$still,type="n",ylim=c(0.001,0.005),xlab="Year", ylab="stillbirth rate",

bty="l",xaxt="n")

# Add line indicating the policy changes

#abline(v=37,lty=2)

rect(37,0.001,145,0.006,col=grey(0.9),border=F)

# plot the observed rate for intervention period

points(smoke$still,cex=0.7)

lines(smoke$still,cex=0.7)

#specify the x-axis (i.e. time units)

axis(1,at=0:12*12,labels=F)

axis(1,at=0:12*12,tick=F,labels=2005:2017)

# add a title

title("Stillbirth rate, 2005-2016")

#plot regression line

pred <- predict(model1,type="response")

lines(pred/smoke$n,col=2)

############

#VPB

############

plot(smoke$vpb,type="n",ylim=c(0.005,0.014),xlab="Year", ylab="Very PB percentage",

bty="l",xaxt="n")

# Add line indicating the policy changes

#abline(v=37,lty=2)

rect(37,0.005,145,0.014,col=grey(0.9),border=F)

# plot the observed rate for intervention period

points(smoke$vpb,cex=0.7) #oder mit points

lines(smoke$vpb,cex=0.7)

#specify the x-axis (i.e. time units)

axis(1,at=0:12*12,labels=F)

axis(1,at=0:12*12,tick=F,labels=2005:2017)

# add a title

title("e. very preterm birth")

smoke$vpbn <- smoke$vpb * smoke$n

library(MASS)

model2 <- glm.nb(vpbn ~ offset(logn) + t + level + slope, data=smoke, link=log)

summary(model2)

1 - pchisq(summary(model2)$deviance,

summary(model2)$df.residual) #better fit

par(mfrow=c(1, 2))

acf(residuals(model2))

acf(residuals(model2),type='partial') #autocorrelation still present

#model with autocorrelation adjustment

predictors <- cbind(intercept <-(rep(1, dim(smoke)[1])),

slope <-smoke$slope, level <- smoke$level, t <-smoke$t)

colnames(predictors) <- c("intercept", "slope", "level", "t")

model4 <- glarma(smoke$vpbn, predictors, offset = smoke$logn, type = "NegBin", phiLags = c(3,5,11,12))

#final model

summary(model4)

acf(residuals(model4))

acf(residuals(model4),type='partial')

#plot regression line

coef(model4)

predict <- exp(smoke$logn + coef(model4)$beta[1]

+ smoke$t*coef(model4)$beta[4]

+ smoke$level*coef(model4)$beta[3]

+ smoke$slope*coef(model4)$beta[2])

lines(predict/smoke$n,col="red")
